# Optimizing CPAP Adherence in Obstructive Sleep Apnea: A Narrative Review of Practical Strategies for Everyday Clinical Care

**DOI:** 10.3390/healthcare14152328

**Published:** 2026-08-01

**Authors:** Caterina Antonaglia, Antonio Fabozzi, Alessia Steffanina, Sara Soave, Paola Confalonieri, Ambra Nicolai, Federica Olmati, Arianna Sanna, Nahuan Pena, Barbara Ruaro, Francesco Salton, Matteo Bonini, Marco Confalonieri, Paolo Palange

**Affiliations:** 1 Pulmonology Unit, Department of Medical Surgical and Health Sciences, Hospital of Cattinara, University of Trieste, 34149 Trieste, Italy; sara.soave@asugi.sanita.fvg.it (S.S.); paola.confalonieri@asugi.sanita.fvg.it (P.C.); barbara.ruaro@asugi.sanita.fvg.it (B.R.); francesco.salton@asugi.sanita.fvg.it (F.S.); marco.confalonieri@asugi.sanita.fvg.it (M.C.); 2 Pulmonology Unit, Department of Public Health and Infectious Diseases, Policlinico Umberto I, “Sapienza” University of Rome, Viale del Policlinico, 155, 00161 Rome, Italy; antonio.fabozzi@uniroma1.it (A.F.); a.steffanina@policlinicoumberto1.it (A.S.); a.nicolai@policlinicoumberto1.it (A.N.); f.olmati@policlinicoumberto1.it (F.O.); a.sanna@policlinicoumberto1.it (A.S.); matteo.bonini@uniroma1.it (M.B.); paolo.palange@uniroma1.it (P.P.); 3 Independent Researcher, 34149 Trieste, Italy; nahuanuni91@gmail.com

**Keywords:** obstructive sleep apnea, continuous positive airway pressure, adherence, obstructive sleep apnea treatment, personalized medicine

## Abstract

**Background:** Continuous positive airway pressure (CPAP) is the primary treatment for obstructive sleep apnea (OSA). Despite improvements in CPAP technology and management, adherence to therapy remains one of the main challenges to be addressed. **Methods:** We conducted a narrative review through PubMed (1995–2026). Studies were selected by clinical relevance, methodological quality and expert consensus. **Results:** OSA treatment outcomes are poor when CPAP adherence is defined as four hours per night. The first step in improving adherence is active patient involvement. This involves explaining what OSA is, its consequences, what PAP therapy is, and its potential benefits. The choice of the right mask is the first personalized step in CPAP adherence. OSA phenotype and endotype traits may help personalize treatment in selected patients, although their role in predicting CPAP adherence needs to be studied in greater depth. Problems during the first night and the first month are the main predictors of future adherence. Strategies such as cognitive behavioral therapy or motivational enhancement can improve adherence, especially during the initial period. Long-term adherence can be predicted from the initial period and maintained through scheduled follow-up. Group meetings, telephone calls and telemedicine interactions are also a valid way of improving adherence. **Conclusions:** Patients should initially be educated about how their symptoms are related to sleep apnea and how CPAP treatment could resolve them. The key to improving CPAP adherence is involving patients in personalized treatment with scheduled follow-up, particularly during the initial treatment period.

## 1. Introduction

Continuous positive airway pressure (CPAP) therapy represents the first-line treatment for obstructive sleep apnea syndrome (OSAS), effectively reducing disease severity, daytime sleepiness, systemic blood pressure, and motor vehicle accidents, while significantly improving health- and sleep-related quality of life [1]. Although CPAP efficacy remains otherwise well established, regardless of the site of upper airway obstruction, the long-term benefits for the cardiovascular and metabolic issues have been attenuated by suboptimal adherence [1,2]. Indeed, over the past decades, adherence to CPAP therapy has emerged as one of the central issues in the literature on OSAS, representing the principal treatment limitation. Accordingly, scientific interest in this topic has progressively increased [3,4]. Available evidence indicates that low adherence is a multifactorial phenomenon influenced by physician-related variables, patient characteristics, disease severity and phenotype. The first way to improve adherence is to consider the clinical definition. Adherence is defined as the active and collaborative involvement of the patient in the proposed treatment. For this reason, as we discuss later, active involvement is one of the most important aspects. In the literature, symptoms such as drowsiness, subjective benefit from therapy in the early stages, snoring, and use of a nasal mask are well-established indicators of good adherence, while the oronasal mask, low socioeconomic status, and claustrophobia are examples of factors that are indisputably associated with low adherence [5]. Encouraging adherence is a part of the daily clinical practice of all sleep specialists but is also a challenge. In the last few years, the literature has focused on the main factors able to improve adherence and the different strategies used by clinicians [3]. Recent evidence demonstrated a statistically significant reduction in hospitalizations and emergency room visits in obese OSA patients with good adherence to CPAP compared to poorly adherent ones [6]. Although improvements have been made in CPAP technology and the masks, only 50–60% of patients show long-term adherence to this treatment [4]. This poor adherence to CPAP treatment among a significant proportion of the OSA population highlights the need to develop a more integrated and patient-centered approach. More recently, however, the American Academy of Sleep Medicine (AASM) guidelines systematically emphasized the importance of targeted interventions aimed at improving adherence [7]. The clinical practice guideline for the treatment of OSA emphasizes three fundamental steps in achieving adherence: educational, troubleshooting and behavioral interventions. Within this framework, the present review aims to synthesize the available evidence on the strategies with the strongest scientific support that should constitute the foundation of any CPAP initiation program. In addition, psychosocial interventions, particularly cognitive behavioral therapy for insomnia (CBT-I) in patients with comorbid insomnia and sleep apnea (COMISA), will be discussed as promising but not yet fully standardized approaches [8,9]. More exploratory strategies, such as phenotype- or endotype-guided titration, will also be addressed, given their potential to support a more individualized approach not only to treatment delivery, but also to adherence promotion [10,11]. Personalized medicine in OSA has shown that some patients—for example, due to endotype factors (low arousal threshold) and phenotypic factors (women with insomnia or young adults with insomnia)—have poor adherence to CPAP [12]. For these patients, tailoring adaptation strategies—as has been done for the female “phenotype” using a machine with a dedicated algorithm or a combined approach, such as CPAP + CBT for young adults with insomnia—may be the right way to address the adherence issue through greater personalization of the therapy itself, rather than simply dividing patients into adherent and non-adherent groups [13,14]. In conclusion, the aim of this narrative review is to rethink CPAP adherence with a multifactorial approach that takes into account patient traits, their phenotype, and treatment-related factors, leading to a personalized approach to optimize long-term adherence.

## 2. Materials and Methods

This narrative review was undertaken to provide a comprehensive and clinically oriented synthesis of the contemporary evidence on the factors influencing adherence to continuous positive airway pressure (CPAP) therapy in patients with obstructive sleep apnea syndrome (OSAS), as well as on practical strategies that may improve adherence in routine clinical care. We searched PubMed/MEDLINE (via the PubMed interface) and Google Scholar, covering publications from January 1995 through 20 June 2026; the exact final search date and the full queries are provided in Supplementary Material Table S1. The strategy combined three conceptual blocks (population, interventions, outcomes) using Mesh and free-text terms (e.g., “obstructive sleep apnea", “OSA", “continuous positive airway pressure", “CPAP adherence", “compliance", “telemedicine", “cognitive behavioral therapy", “mask interface", “phenotype", “endotype”); the exact Boolean strings with field tags and any filters, as executed, appear in Supplementary Material Table S1. We applied operational inclusion criteria prioritizing studies on predictors of CPAP adherence, barriers to sustained use, and clinically actionable interventions; inclusion/exclusion rules (population, study designs, outcomes, time frame, language) are specified below. Priority was given to recent original investigations and meta-analyses; clinically oriented reviews were included when they synthesized actionable evidence, while case reports and non-data commentaries were excluded. Particular attention was paid not only to the identification of factors associated with greater or lower CPAP adherence, but also to the clinical interpretability of those findings. For the purposes of this review, studies and meta-analyses that were limited to a purely descriptive listing of variables associated with adherence were considered less informative than those that offered a broader interpretative framework through which the observed associations could be understood in clinical practice. Accordingly, preference was given to studies that, beyond reporting statistical associations, also explored the potential behavioral, physiological, and practical mechanisms underlying those results. This approach was intended to support a more clinically meaningful discussion of adherence as a multidimensional and dynamic process, rather than as the simple sum of isolated predictors. We included randomized trials, prospective and retrospective observational studies, and meta-analyses of adults (aged ≥18 years) with OSA, evaluating CPAP or auto-CPAP with adherence outcomes (e.g., hours of nightly use, nights of ≥4 h) and/or indirect effectiveness aligned with physiological mechanisms. We excluded in vitro/animal studies, pediatric studies, editorials/letters without primary data, narrative overviews lacking decision-guiding synthesis, single-case reports, conference abstracts without full text and duplicate/overlapping cohorts (retaining the most comprehensive or recent analysis). Two authors independently reviewed titles, abstracts, and full-text articles in order to identify studies of greater clinical and methodological relevance. Eligible designs included randomized trials, prospective and retrospective observational studies, and meta-analyses addressing CPAP adherence, its determinants, and strategies for improvement; narrative reviews were considered when they added decision-guiding synthesis. No formal protocol was prospectively registered for this review. In addition, given the narrative nature of the article, no PRISMA-based methodology, SANRA framework, or formal standardized quality assessment tool was applied. Selection and interpretation were based on prespecified relevance, cross-study consistency, and clinical applicability; study-level decisions and reasons are documented in Supplementary Material Table S2. A PRISMA-style flow with counts (identified, deduplicated, screened, full-text assessed, excluded with reasons, included) is provided in the Supplementary Materials. Any differences in interpretation were discussed among the authors and resolved by consensus. This article is based exclusively on previously published studies and does not include any new studies involving human or animal subjects conducted by the authors. In addition to a structured keyword search on adherence, we deliberately re-examined each clinically salient dimension—pathophysiology, mask selection, technical refinements, CPAP versus auto-CPAP comparisons, and adherence in women with OSA—to identify evidence most capable of guiding diagnostic, therapeutic, and follow-up decisions, capturing both direct adherence outcomes and indirect effectiveness aligned with physiological mechanisms.

## 3. Results

### 3.1. CPAP Adherence: Definition, Time Frame and Limitations

In 2009, the Centers for Medicare & Medicaid Services adopted a requirement for a minimum 4 h of positive airway pressure (PAP) use for 70% of nights, or 21 days in a consecutive 30-day period, to continue medical coverage for PAP therapy [15]. The use of an economic rather than a clinical definition is the first CPAP adherence issue to be fixed. Indeed, this is commonly used for reimbursement or coverage purposes but, although it is not clinically ineffective for all patients, the reduction of the AHI could be only partial [16]. A few hours during the night may not improve symptoms, quality of life or reduce cardiovascular risk, with a subsequent reduction in patient motivation. Continuous positive airway pressure (CPAP) adherence using an “≥4 h on ≥70% of nights” metric represents an administrative or reimbursement threshold used by payers and device monitoring programs and does not, by itself, define clinically meaningful treatment adequacy. First, this threshold has been criticized as being too low by some and too strict by others for patients who may sleep less and benefit even less from 4 h of sleep [17]. Second, it also appears in the research literature as a reporting convention that enables comparability across studies. Third, clinically meaningful CPAP use is better conceptualized as the duration needed to achieve patient-centered outcomes (symptom relief, functional improvement) and risk modification (cardiovascular and metabolic), which may exceed administrative minima for many patients and may vary by phenotype and sleep pattern. Although evidence supports a dose–response relationship between nightly CPAP duration and clinical benefit, this relationship should be articulated carefully to avoid overgeneralization [3]. Observational and experimental data indicate that limited nightly use can reduce disease burden, yet the magnitude of benefit often scales with longer wear times, daytime sleepiness improves after 7 h of sleep, while 7 h and 30 min is the medium time necessary to reach a good performance status [7,18]. This range could also be different for shorter or longer sleepers and vary during the lifespan. Certainly, a cut-off value is necessary in the literature to quantify the adherence and to compare different reports. An OSA patient who removes the mask after only the first 4 h of sleep will not treat the deeper desaturations, the more severe and longer events in Rapid Eye Movemements (REM) sleep that prevail in the second stage of sleep, and this is associated with an increase in cardiometabolic risk [19,20]. In addition, recent evidence underlines that patients with REM-related OSA, with more than 50% of events during REM sleep, demonstrated significantly lower nightly CPAP use, in particular, in long-term adherence compared with patients with non-stage-specific OSA [21]. CPAP efficacy, or dose–response effect, suggests that the ineffectiveness of CPAP in terms of cardiovascular and metabolic health in some trials is partly due to poor or non-optimal adherence [1,22]. Heterogeneity in sleep duration, OSA phenotype, comorbidities, and patient priorities further caution against a one-size-fits-all adherence target. Short sleepers may accrue meaningful benefit at lower absolute nightly hours than long sleepers, though—consistent with dose–response principles—additional hours commonly produce additional gains. Conversely, some patients with severe, non-stage-specific OSA or substantial cardiometabolic risk may require near–full-night use to meaningfully modify outcomes. Real-world data also suggest that adherence patterns and therapy persistence vary across OSA phenotypes and patient profiles, with implications for how thresholds are met—or not met—in practice [23]. Another aspect is the definition of adherence compared to compliance. The term adherence itself underlines the necessity of active and collaborative treatment acceptance, including a self-efficacy meaning, hence no exclusive terminological difference with the most passive compliance. Adherence is also different from acceptance. Acceptance is defined as willingness to purchase and use a CPAP device, and it could be related to financial issues, social stigma or intolerance to CPAP or preference for another therapy [24]. In sleep medicine, the time issue is also important, as it defines short- and long-term adherence. Long-term adherence refers to different time horizons (3, 6 or 12 months), while shorter adherence refers to the first month of CPAP treatment and strongly influences the following period [25].

### 3.2. Patient-Related Factors

**Figure 1:**
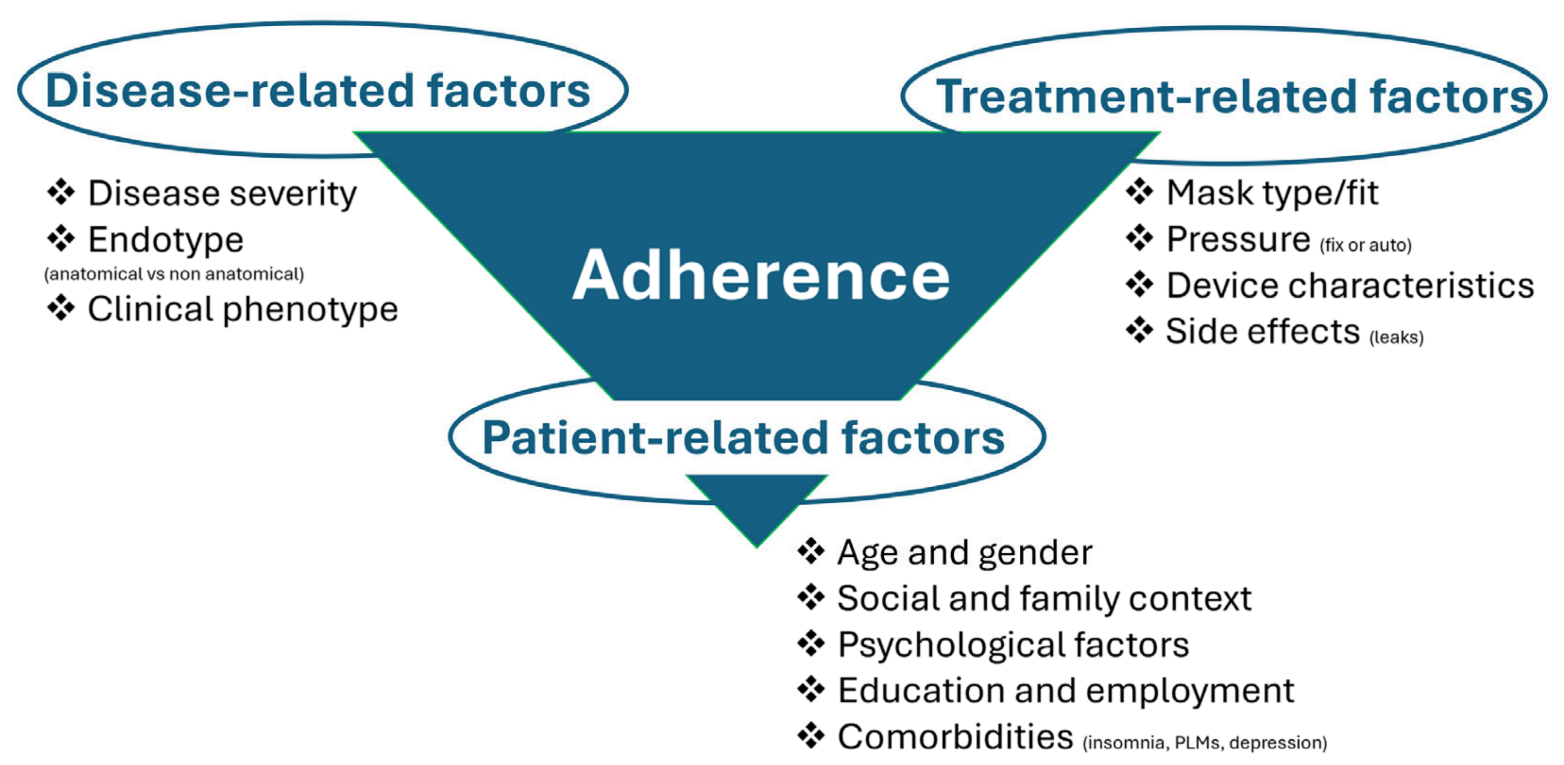
Interplay of disease-, treatment-, and patient-related factors shaping. Continuos Positive Airway Pressure (CPAP) adherence; patient-related factors are central.

We can divide the factors that influence adherence into patient-related factors (sex, gender, age, socioeconomical status, etc.), disease-related factors (endotype and phenotype), and treatment-related factors (like mask or PAP) (Figure 1). The data regarding sex are currently controversial, as some studies have shown that adherence to CPAP among women is lower than that of men, while other studies have shown the opposite [26,27]. Another recently published article suggests that women under 50 experience the largest decline in CPAP adherence. These findings emphasize the need for strategies tailored to different age groups and sexes to encourage long-term engagement with PAP therapy [28]. Data on sex differences in CPAP adherence remain inconsistent. One possible explanation is that earlier studies did not adequately account for relevant comorbidities that are particularly frequent in women, such as COMISA, depression, restless legs syndrome, and other sleep-related or psychiatric conditions, all of which may substantially influence treatment acceptance and long-term use. In addition, the apparent effect of sex may be partly confounded by social and behavioral factors that have changed over time, although this hypothesis has not been formally demonstrated. What appears more certain is that women with OSA represent a heterogeneous group, with phenotypic differences that remain less well characterized than in men, as also suggested by the ESADA study. Therefore, discussing CPAP adherence in women as a single, uniform entity is likely an oversimplification [29]. Recent findings suggest paying attention to the relationship with the bed partner. Concerns about the impact of CPAP on physical closeness, communication, and sexual intimacy are frequently cited by patients as barriers to regular use [30]. Adequate support is necessary to improve CPAP adherence, but on some occasions, the bed partner is an obstacle to use of the device [31]. A few studies suggest that a good understanding of what CPAP is could help couples to become more familiar with the device [31]. On the contrary, other patients report that the equipment used can interrupt the couple’s intimacy. This has a negative effect on both the relationship and adherence to therapy, creating a vicious circle that worsens both [32]. Data on the impact of age on treatment adherence, however, consistently show a biphasic pattern: the number of hours of use per night increases gradually up to the age of 75–80, after which there is a rapid decline in night-time use [33]. Comorbidities also influence CPAP adherence. Comorbid insomnia and obstructive sleep apnea (COMISA), the condition in which OSA and insomnia coexist, is associated with a greater burden of symptoms and lower CPAP usage compared to patients with OSA alone [34]. However, randomized clinical trials have shown that the use of cognitive behavioral therapy for insomnia (CBT-I) prior to CPAP significantly increased CPAP adherence in COMISA patients [35,36]. For this reason, it is necessary to recognize and treat insomnia first, or at the same time as starting CPAP treatment. Starting CPAP therapy in OSA patients suffering from depression, a quite common comorbidity (around 35% according to a large systematic review), could be challenging [37]. Real-world data show that the presence of depression is associated with reduced long-term adherence to CPAP and also fewer hours of APAP use during the first week of auto titration [38,39]. Clinically significant anxiety, with a prevalence of around 32% in OSA, is also associated with a reduced number of hours of night-time CPAP use, although anxiety scores improve significantly in OSA patients adhering to CPAP therapy [37,38]. Restless legs syndrome (RLS) and periodic limb movements (PLMs) are two other common comorbidities in OSAS (around 20% for RLS and around 14% for PLMs) that could interfere dramatically with the CPAP adherence [40,41].

### 3.3. Disease-Related Factors

Phenotype- and endotype-informed approaches may help explain inter-individual variability in CPAP acceptance and sustained use. Patients with the sleepy phenotype are generally more likely to adhere to the treatment because their quality of life improves significantly using CPAP [42]. Snoring is another symptom that disappears immediately with CPAP, and this has a strong impact on adherence due to the beneficial effect on the patient and his/her bed partner [43]. Non-sleepy, older with comorbidities and female are three phenotypes less likely to adhere to the treatment [44]. One common misconception is that patients with mild OSA are less likely to adhere to CPAP therapy. For example, patients with a low arousal threshold could experience significant sleepiness [45]. CPAP therapy improves their symptoms. For this reason, patients with mild OSA who are experiencing symptoms may be more likely to adhere to treatment than those with severe OSA who are not experiencing symptoms. Clearly, we cannot use AHI to predict a patient’s adherence to CPAP treatment. Recent evidence suggests that a “one size fits all” approach is not appropriate for OSA, as the pathophysiology of upper airway collapse can differ from patient to patient [46]. The anatomical susceptibility to upper airway collapse varies among patients with obstructive sleep apnea (OSA), leading to differences in the efficacy of continuous positive airway pressure (CPAP) therapy between individuals. In some patients, the predominant anatomical factor is associated with greater CPAP efficacy. In other patients, however, this factor is less prominent, and other non-anatomical traits, such as high ventilatory control instability (high loop gain), a low arousal threshold, and poor upper airway muscle responsiveness, may contribute to OSA pathophysiology and limit CPAP efficacy and tolerance [47]. On the other hand, patients with a low arousal threshold may report frequent arousals during pressure variations in auto-adjusting PAP [48]. A low arousal threshold is characterized by a less negative endo-esophageal pressure threshold that induces arousal. In this patient, when the upper airway begins to close, as in a flow limitation, with minimal negative endo-esophageal pressure, the patient wakes up. The pCO_2_ value becomes unstable, as does sleep, and N3 sleep decreases. The patency of the upper airway also decreases. In short, a low arousal threshold refers to a patient who is “easy to wake up” and, for this reason, is more susceptible to changes in pressure treatment levels. If the non-anatomical trait prevails in these patients, CPAP could not be the first choice of treatment [49]. In patients with severe OSA, a high loop gain is more common and may compromise CPAP adherence, as suggested by the occurrence of treatment-emergent central sleep apnea (TECSA), at least in the first period of treatment. Patients with high loop gain exhibit an exaggerated ventilatory response to even mild or moderate changes in CO_2_, which amplifies respiratory instability. In such cases, using a narrower pressure range during auto-CPAP titration or opting for fixed-pressure CPAP may be beneficial [50]. Although no dedicated algorithm has yet been implemented for patients with OSA and high loop gain, this represents a promising area for personalized medicine. Expiratory pressure relief (EPR) can indirectly benefit patients with high loop gain by lowering expiratory pressure, which reduces mean airway pressure and helps avoid excessive CO_2_ washout that can trigger ventilatory instability. By decreasing respiratory effort—particularly at higher pressures (>12 cm H_2_O)—EPR may also reduce arousals in these patients, who often have a low arousal threshold, thereby dampening the instability cycle. However, in cases of very high loop gain, EPR alone may be insufficient; more tailored settings or advanced ventilatory modes may be needed. On the other hand, it is also possible that when EPR is used in auto-adjusting devices, the reduction in expiratory pressure is associated with a potentially increased airflow resistance that might be sensed by the machine, leading to an increase in therapeutic pressure with a consequent treatment intolerance and poor adherence [51]. However, the response to expiratory pressure relief across different endotypes remains largely unexplored, and it should not be treated as a minor detail when discussing adherence. For such patients, in-lab manual titration is often the best approach to identify a stable setup that controls obstruction without provoking exaggerated ventilatory responses [52]. However, current evidence does not support using endotypes to predict CPAP adherence, due to the absence of statistically significant differences for adherence between endotypes in pilot studies [38]. Validated tools and workflows for routine clinical endotyping are not yet available, and evidence linking specific endotypes (e.g., low arousal threshold) to reproducible improvements in adherence across unselected populations remains limited. At present, these strategies should be regarded as promising and hypothesis-generating, most appropriate for specialized centers or research protocols, and they require prospective validation before incorporation into everyday clinical care. Although the standard method to understand the main pathophysiological trait is the polysomnographic trace in a sleep research laboratory, certain polysomnographic characteristics in clinical PSG could suggest the prevalent endotype [49,50]. An increased upper airway collapsibility (high Pcrit) or the anatomical factor prevalence could be estimated in clinical routine as a high apnea/hypopnea ratio (i.e., greater predominance of apnea versus hypopnea is associated with a more collapsible airway), or could be correlated with a higher therapeutic CPAP level (i.e., higher therapeutic CPAP requirements are associated with a more collapsible airway) [53,54,55]. In patients with impaired pharyngeal dilation muscle function, airflow becomes progressively worse during respiratory events. A prevalent REM OSA suggests that the dilator muscles are capable of restoring airflow to the airway, narrowing during non-REM sleep when there is sufficient drive (which is reduced in REM). If non-REM and REM AHI are similar, this would be consistent with poor pharyngeal dilator muscle function throughout sleep [56]. Concerning a low arousal threshold, this endotype can be easily estimated from just standard polysomnography outputs with high sensitivity and specificity, as follows: Low respiratory arousal threshold score with AHI < 30 events/h sleep + o Nadir SpO_2_ > 82.5% + o Fraction of hypopneas > 58.3%, with a score ≥ 2 predicting a low respiratory arousal threshold (with a sensitivity = 80.4%, specificity = 88%, positive predictive value = 87%, negative predictive value = 81%) [49]. In more clinical terms, a milder presentation characterized predominantly by hypopneas and/or flow limitation, with brief events and minimal oxygen desaturation, is suggestive of this endotype [57].Unstable respiratory control or high loop gain could be easily identified as the main factor by reviewing the polysomnography record and looking for cyclical patterns of breathing including large ventilatory overshoots in airflow following arousal/respiratory events [50,58]. Evidence of central/mixed events/elevated central apnea indices would also be consistent with high loop gain, as would evidence of cardiovascular disease (i.e., heart failure) or increased OSA severity in non-REM sleep. The effort to recognize these characteristics—pending the development of validated methods for their identification—is not merely an exercise, because guidance on which therapeutic approaches work and which do not, depending on the endotype, is already well-established in the literature [11]. Observational and modelling data suggest that a low arousal threshold may contribute to CPAP intolerance in some patients, yet confirmatory prospective studies demonstrating adherence gains with endotype-tailored strategies are still needed [34]. To avoid over-translation while preserving clinical utility, we clarify three practical tiers: First of all, routine clinical clues from standard PSG/home sleep apnea tests, which we illustrate for each predominant endotype: apnea-predominant events or higher therapeutic CPAP levels suggesting increased collapsibility (anatomical factor); coexisting or mixed central events, periodic/waxing–waning breathing, or Cheyne–Stokes–like instability suggesting high loop gain; the Edwards composite criteria (AHI < 30 events/h, nadir SpO_2_ > 82.5%, fraction of hypopneas >58.3%; score ≥2 indicating a low arousal threshold) to screen for low arousal threshold; and REM-dominant OSA as a clinical marker of reduced dilator muscle responsiveness [19,49,58]. Second, specialized-center assessments that estimate traits from routine full PSG using validated model-based inference, notably the mathematical framework described by Terrill et al., which computes ventilatory-control endotypes from standard signals without invasive instrumentation [59]. Finally, research-level measurements requiring full laboratory protocols, including esophageal pressure monitoring and CPAP platforms permitting dial-down pressure CPAP manipulations (and, where applicable, targeted gas perturbations), which remain outside the practical scope of this review [59,60]. Phenotype/endotype-informed strategies to improve adherence are discussed as promising and research-oriented, not yet feasible for routine care, and requiring prospective validation.

### 3.4. Device-Related Factors

The choice of an appropriate interface represents a key point for treatment adherence. An inadequate size may result in discomfort or excessive leaks. Patients generally prefer nasal masks and nasal pillow interfaces for their lower contact surface and greater comfort [61]. Current evidence supports that nasal interfaces are associated with better adherence, improved comfort, and lower leak rates compared with oronasal masks [62]. A nasal mask also reproduces natural breathing, whereas oral breathing only minimally contributes to sleep breathing pattern [63]. Indeed, oral breathing is associated with increased upper airway resistance and reduced upper airway stability [64]. For these reasons, the use of oronasal masks may be counterproductive by promoting posterior tongue collapse and increasing pharyngeal collapsibility [64]. Moreover, oronasal masks typically require higher PAP compared with nasal interfaces [65]. Between nasal masks and nasal pillows, available evidence suggests comparable efficacy in terms of residual AHI and sleep architecture, with only minimal differences in adherence [66,67]. Interface selection should be individualized and reassessed early. Indeed, the nasal mask advantage is not universal and depends on patient factors and clinical context. Oronasal masks remain appropriate in selected situations, notably in patients with persistent relevant nasal obstruction or predominant mouth breathing despite targeted optimization (e.g., nasal therapies, humidification, chin strap). However, the observation of oral breathing during sleep, by itself, is not an indication to select an oronasal mask. Our practical approach is to start with a nasal interface when reasonable, actively manage nasal symptoms and humidification, and switch interface if leaks, discomfort, or efficacy issues persist. This stepwise, patient-centered strategy avoids categorical claims while preserving the clinical importance of nasal masks in routine care. Approximately 15% of OSA patients report nasal obstruction, without any correlation between OSA severity and nasal obstruction severity [66]. Increased nasal resistance does not directly cause OSA, but can increase upper airway resistance, leading to oral breathing predominance and increased pharyngeal collapsibility [68]. As demonstrated by Sugiura T et al., increased nasal resistance before CPAP treatment predicted CPAP non-adherence [69]. For these reasons, we need to improve nasal patency before the first CPAP trial. A complete evaluation including the investigation of nasal symptoms, history of allergic rhinitis, chronic congestion or prior nasal surgery is necessary. Saline irrigations and intranasal corticosteroids may be particularly effective before CPAP titration in patients with inflammatory conditions [70]. Another crucial point of CPAP adherence is the therapeutic PAP choice. Nowadays, home-based PAP auto-titration represents the most widely adopted and cost-effective approach [52,71]. Home-based PAP auto-titration starts with an initial period where PAP may be suboptimal, potentially leading to residual respiratory events, flow limitation or snoring [52]. These factors may negatively affect early adherence, highlighting the importance of appropriate patient counselling to set expectations during this phase [51]. Reducing these residual events by controlling pressure settings is associated with improved CPAP adherence and increased comfort [72]. Importantly, the first 2–4 weeks of therapy are a critical period for future CPAP adherence [73]. During this phase, suboptimal titration and PAP-related discomfort may significantly contribute to low long-term adherence, highlighting the importance of early PAP optimization and initial close follow-up [53]. As concerns the comparison between APAP and fixed CPAP in terms of long-term adherence, the issue remains a matter of debate. The currently available evidence has not demonstrated a clear advantage for either CPAP or APAP in terms of adherence, daytime sleepiness, or quality of life [74]. Regardless of the strategy used, a careful manual analysis of the downloaded data remains essential both during titration and during follow-up [7,75]. The aim of the titration phase is to set the correct pressure to correct all respiratory events (snoring, flow limitations, apnea and hypopnea) with minimal pressure and without leaks. It is also necessary to ensure sufficient CPAP use and efficacy in different sleep stages, sleep positions and night-time fluctuations. To achieve this, manual data downloads performed by attentive, knowledgeable staff with appropriate familiarity with the devices used are necessary [76].

### 3.5. The Patients’ Perceptions

CPAP and OSA patients’ perceptions represent a key point for obtaining good long-term adherence. The patient is unaware of what happens during sleep. Daytime symptoms are not always immediately attributable to the sleep disorder. Awareness among the general public regarding obstructive sleep apnea syndrome and its consequences is limited or even nonexistent. For this reason, the guidelines of the AASM recommend, first and foremost, that patients be properly informed as early as their first visit [7]. Studies have shown that explaining the syndrome during the initial visit increases patient awareness and, consequently, adherence [55,56]. Effective communication should include a clear explanation of obstructive sleep apnea, its clinical consequences, and the expected benefits of CPAP therapy. Personalizing this discussion, by linking the patient’s reported symptoms to OSA and explaining how CPAP may alleviate them, can enhance treatment acceptance. Accurate information for the patients about their conditions led to a 1 h increase in daily CPAP use [77]. Many patients are not aware that symptoms such as nocturia or morning headaches may be related to sleep apnea, and increasing this awareness may improve engagement. Another simple intervention to improve therapy adherence may be reviewing polysomnographic data (e.g., oxygen desaturation and airflow tracings), which has improved both follow-up attendance and long-term adherence [78]. Overall, these findings highlight the central role of the initial approach to CPAP therapy, underscoring the importance of patient-centered communication during both the first encounter and the titration phase.

### 3.6. Follow-Up and Practical Approach for Early Solution of Problems

**Scheme 1:**
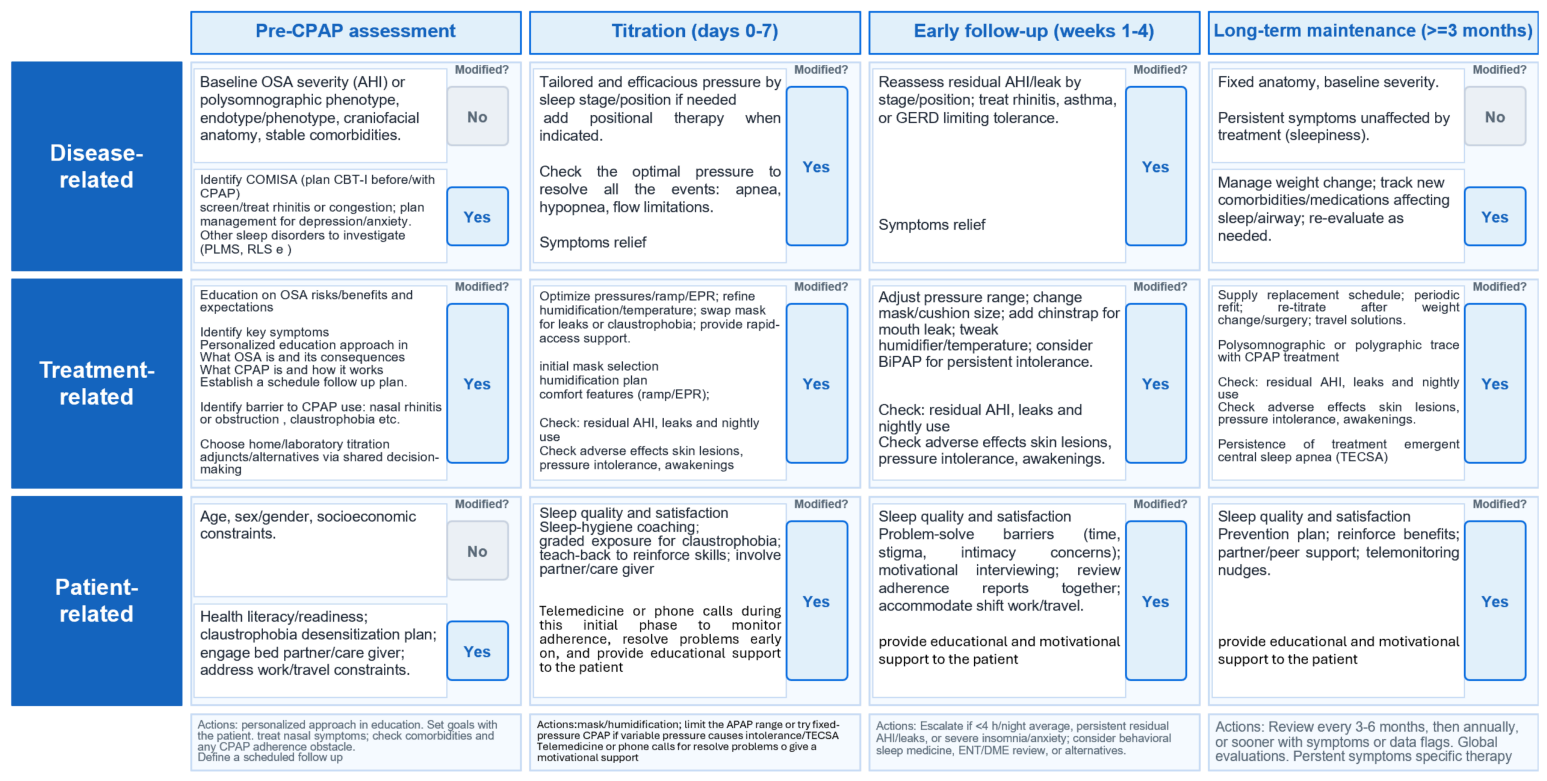
Factors influencing CPAP adherence: what to address, when, and what’s modifiable.

**Figure 2:**
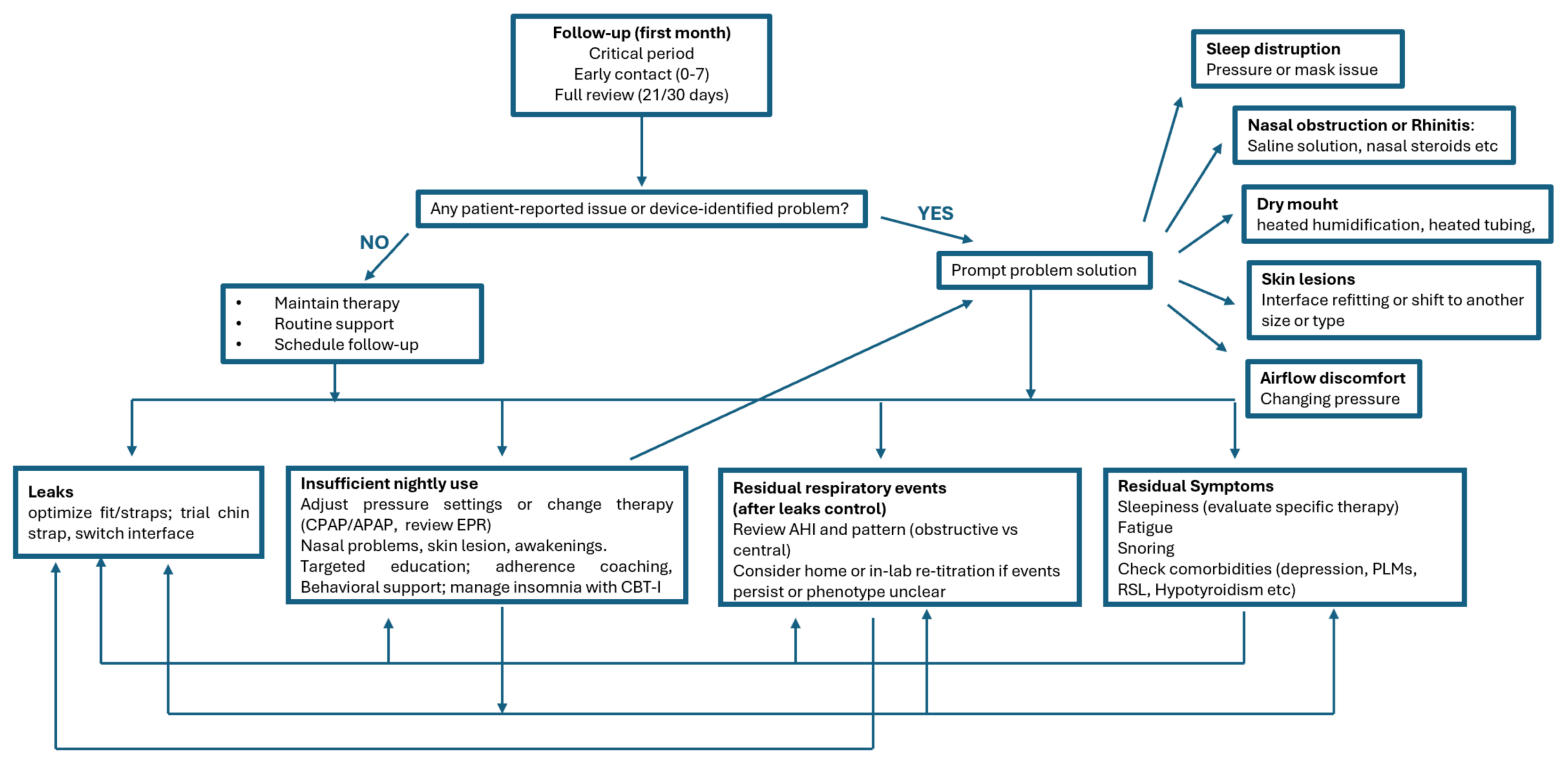
Stepwise CPAP follow-up: at each visit, check leaks, usage hours, and residual symptoms; optimize mask fit and pressures; if adherence is reduced, implement motivational strategies; if residual sleepiness persists, rule out comorbidities (depression, PLM, medications) and consider pharmacologic therapy.

During the pre-CPAP period or baseline, a rapid evaluation of adherence predictors and barriers is performed first. In particular, comorbidities such as insomnia, depression, anxiety and other sleep disorders are investigated. If insomnia is present, CBT-I therapy may need to be started before CPAP assessment [79]. Nasal obstruction or allergic rhinitis are also taken into account, and specialist evaluation or specific treatment may be required [80]. Immediately prior to titration, a careful review of the home sleep apnea testhy or polysomnographic recording may be helpful to characterize the sleep architecture and pattern, identify the endotype, and detect the presence of REM-predominant OSA or a more severe oximetric profile that warrants close monitoring once obstructive apneas have been corrected [19,57,58]. An approach based on the Failure and Effects Analysis (FMEA) framework could be useful in this sense. The FMEA is a prospective risk-management strategy already used across several patient safety domains, such as radiotherapy and blood transfusion services [81,82]. It consists of prospectively mapping the CPAP initiation process to identify patient-specific failure modes (e.g., anatomical predisposition to oral leaks, claustrophobia, poor proficiency with technological devices), with the objective of ranking them by severity, occurrence and detectability to take preventive measures before therapy initiation. As previously acknowledged, this framework has not yet been formally applied to CPAP initiation pathways, and its potential utility in this context should be regarded as conceptual and hypothesis-generating rather than evidence-based. First-line approach: predict who is at risk, then prevent. Table 1A reports prototypical failure modes and corresponding pre-CPAP mitigations derived from this framework. Start with comorbidities that threaten adherence. A depressed patient may appear non-adherent initially; explain that benefits (including mood improvement) and auto efficacy often accrue over time, keep close follow-up, and make them feel supported and central to care [39]. A patient with claustrophobia could need a gentler pressure profile (ramp/EPR), a smaller/lower-profile interface, and graded in-clinic exposure to CPAP [52]. When the patient’s pattern is not helpful, co-create goals so they become an active partner in therapy. Prevent dry mouth, in general, more likely with nasal obstruction, habitual oral breathing, or severe OSA. Start heated humidification from initiation; choose the right mask and pressure—note that an oronasal mask is not always required [83]. For these symptoms, but also for any adverse event, set expectations that they may occur in the first days before therapy, can be useful. Another proactive strategy could be applied for phenotype or endotype. Pressure strategy for lower-adherence endotypes: for example, narrow-range auto titration or fixed pressure for low arousal threshold or high loop-gain phenotypes [23]. Begin with lower starting pressures when appropriate. Pair with cognitive-behavioral strategies; explain that early tolerance may be limited but improves. Plan longer, denser follow-up with more touchpoints. It can be “normal” at first to remove the mask in the last hours of the night, but doing so reduces efficacy—encourage full-night use as tolerance grows. Because patient factors and solutions are highly individualized, a one-size vad mecum is unrealistic. However, a careful baseline review of comorbidities and PSG/PG patterns, an in-clinic CPAP trial, a proactive conversation, and anticipation of common side effects provide a solid starting point for active management of early failure signals. The prevailing literature largely reflects a Safety-I lens, defining success by the absence of failure (e.g., explaining why 40–50% of patients discontinue or underuse CPAP). Complementing this view, a Safety-II perspective asks why things go well and how successful performance is maintained under varying conditions [84] Rather than characterizing only non-adherent cohorts, we emphasize studying patients with complex, high-risk profiles (e.g., severe claustrophobia, low arousal threshold, comorbid insomnia) who nonetheless achieve and sustain long-term adherence. By characterizing how these individuals adapt, self-correct, and navigate system constraints, we can derive resilient, transferable strategies to embed in peer-support models, onboarding education, and follow-up pathways. For claustrophobic patients, a short, graded in-clinic desensitization combined with a smaller nasal interface and ramp/EPR from the first night enabled rapid acclimation; by week 2, many sustained at least 4 h per night. When low perceived benefit was the primary barrier, anchoring expectations to the patient’s chief complaint (e.g., morning headaches) and providing early telemonitoring feedback fostered a tangible sense of progress and led to a steady increase toward full-night use. In patients with COMISA, delivering a brief CBT-I alongside CPAP, paired with a fixed bedtime routine and closer follow-up, reduced sleep-onset latency and stabilized adherence over the first weeks [85]. Coupling Safety-I and Safety-II. The accompanying pre-CPAP FMEA table prospectively identifies high-risk failure modes and assigns pre-emptive mitigations (Safety-I), while the narrative examples show how high-risk patients succeed and how these resilient adaptations can be codified into peer-support and educational pathways (Safety-II). Together, the components of this dual lens provide a more robust basis for improving real-world CPAP use during the critical early window when adherence trajectories are most malleable. Decisions about treatment at this stage are shared with patients regarding home or laboratory titration, the latter being considered when comorbidities are present. Personalized education about disease and CPAP treatment is required and strongly recommended. The first step in CPAP treatment is to arrange a clear follow-up schedule with the patient. The titration phase through days 0 to 7 depends on the outpatient clinic workflow. Early follow-up represents one of the most effective interventions for improving long-term PAP adherence and should be regarded as a dynamic, patient-centered adaptation process rather than simply the identification of an effective therapeutic pressure [5]. During the titration phase, the patient has a first, guided exposure to the mask and device, which—as guidelines suggest—must be accompanied by structured patient education [7]. Once the interface is selected, instruction on correct placement can be delivered with simple tools (e.g., practicing in front of a mirror; step-by-step photos or a short video on the patient’s phone) up to more advanced options (e.g., augmented/virtual reality modules), as well as app- or video-based reinforcements [86]. In more complex cases, caregiver education is advisable. Over the first months, this educational component may require periodic retraining [44]. It is essential that the staff teaching mask placement and device use are appropriately trained. Although patient-side operation of CPAP is straightforward, helping the patient feel at ease with the device, adjusting ramp pressure or humidification as needed, explaining feedback provided by the device, and using connected apps that visualize therapy feedback to reassure the patient are integral to the educational process. Framing this as a collaborative exercise fosters the active, willing engagement that underpins adherence [3]. Less technical but equally important elements—anxiety management, self-efficacy building, motivational reinforcement, and cognitive-behavioral or motivational therapies—should be selected according to local resources and context; no single strategy has proven universally superior [4,87], A consistent finding, however, is that motivational reinforcement—even as simple as a brief phone call—can support and improve patient adherence [88]. Current guidelines emphasize that the first days and weeks of therapy are crucial for identifying and correcting factors that may compromise treatment acceptance before they lead to treatment discontinuation. When PAP initiation is performed on an outpatient basis, an acclimatization period with auto-adjusting PAP may allow optimization of the pressure range, minimum and maximum pressure settings, and ramp activation and duration according to the patient’s sleep-onset latency, as well as overall treatment tolerance before defining long-term settings [7]. The most commonly reported side effects within the first month include dry mouth and nasal symptoms, such as congestion or mucosal irritation [89]. Nasal or oral dryness may improve with heated humidification and, when appropriate, heated tubing, whereas mask-related skin lesions usually respond to interface refitting, reduction of strap tension, interface replacement, or protective dressings. Additional issues may include mask leaks, skin lesions related to the interface, airflow-related discomfort, and sleep fragmentation [90]. In these cases, a careful management of mask fitting, circuit leaks and air humidification may improve patients’ adherence [91]. Pressure intolerance can often be improved by optimizing pressure settings, ramp duration, or expiratory pressure relief (EPR), while persistent treatment-emergent central sleep apnea may require closer follow-up and, in selected patients, modification of the ventilatory strategy [7]. During each follow-up visit, clinicians assess the following objective metrics: nightly use (hours per night; percentage of nights with ≥4 h of use), residual AHI (with obstructive vs. central breakdown), leaks (median and 95th percentile), pressure profile (median and 95th percentile), flags for treatment-emergent central apneas, and symptoms of relief or persistence, as well as any adverse effects such as residual sleepiness, insomnia/sleep fragmentation, nasal/oral dryness, aerophagia, mask-related skin issues and patient-reported intolerance. Titration pressure selection or follow-up based on automated APAP reports must not overlook essential steps such as downloading device memory data, analyzing flow curves, detecting unintentional leaks and verifying the resolution of respiratory events during sleep. Manual data download during both the titration and follow-up periods is another factor that significantly increases PAP adherence [92]. Mask, size and cushion adjustments should be made to mitigate leaks. If TECSA are present or pressure variability is poorly tolerated, the APAP range should be narrowed or a fixed CPAP trial conducted [91,93]. Comorbidity-targeted co-interventions (weight management, positional therapy and insomnia treatment) are different strategies to take into account. If there are persistent residual events with high treatment pressure, a shift to BiPAP or EPR may be required, but a significative increase in adherence has not been demonstrated [52]. Provide brief adherence support in the form of coaching and troubleshooting. Another important aspect of each phase is the evaluation of clinical outcomes, such as residual symptoms, blood pressure, cardio-metabolic comorbidities and bed partner feedback regarding snoring and awakenings. After excluding secondary causes of residual sleepiness, the persistent symptoms can be managed pharmacologically. Although initiating a specific wake-promoting therapy has not been shown to improve treatment adherence, leaving the patient with residual sleepiness worsens quality of life and undermines perceived treatment efficacy, thereby becoming a driver of non-adherence [94,95]. Educational and behavioral interventions have shown improvements in adherence. Continuous support with telemedicine, telephone follow-up, group-base interviews and cognitive-behavioral therapy may improve CPAP adherence by enhancing patients’ engagement [96,97]. The patient should first be re-evaluated shortly after starting CPAP therapy. Depending on center characteristics and workflow—for example, the use of telemedicine—the timing of the first visit may vary. Where telemedicine is available, day-to-day remote review of PAP downloads enables rapid detection of poor adherence, excessive leaks, or persistent residual events and timely corrective actions, allowing for a longer initial interval. In settings without telemonitoring, a shorter interval is advisable; proactive telephone contact or an early structured follow-up call can similarly identify technical issues, reinforce education, explore concerns, and prompt earlier reassessment when needed. Clinicians should also evaluate emerging conditions during the initial CPAP phase—such as insomnia, depressive symptoms, or sleep-related movement disorders—as these can precipitate CPAP interruption [98,99]. Finally, follow-up intensity should be individualized according to the estimated risk of poor adherence. Patients with anxiety, depression, comorbid insomnia, sleep-related movement disorders, advanced age, cognitive impairment, limited social support, or prior negative experiences with PAP may benefit from a more intensive schedule during the adaptation phase [3,7]. Overall, successful long-term PAP adherence relies on a personalized strategy integrating individualized titration, careful interpretation of PAP waveforms rather than device metrics alone, prompt troubleshooting of side effects, patient-centered education, and a risk-stratified follow-up adapted to both the patient’s needs and the organizational resources of the sleep center. A table with common barriers to CPAP adherence and practical management strategies and a figure with a workflow diagram have been added to this chapter (Table 1, Scheme 1 and Figure 2).

## 4. Discussion

**Figure 3:**
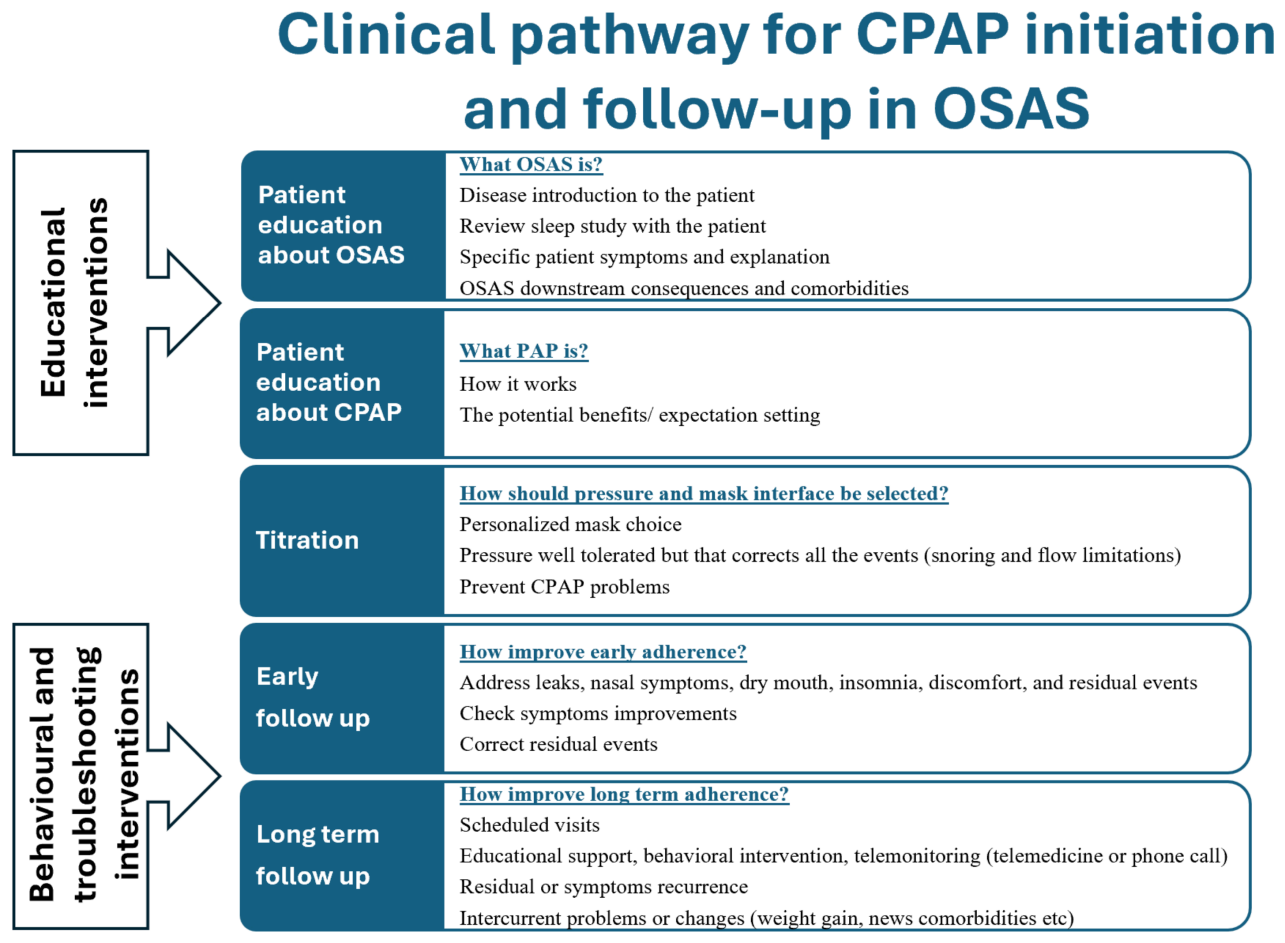
Clinical pathway for CPAP initiation and follow-up in obstructive sleep apnea, outlining education and results review, CPAP introduction and expectations, interface selection with pressure titration, early follow-up to address leaks, symptoms, and residual events, and long-term monitoring via scheduled visits and remote support.

CPAP adherence in OSA is a complex issue that requires a personalized and multidimensional approach. Although CPAP has been shown to be efficacious in improving clinical outcomes, only around half of patients maintain optimal long-term adherence, leading to a critical issue which needs to be prioritized in sleep medicine [103]. In this review, we have highlighted how adherence to CPAP does not depend solely on device- or mask-related factors, but also on patient-related factors such as phenotype and endotype. This issue should be carefully considered when developing a personalized treatment plan for OSA patients. This multidimensional model emphasizes that adherence should be treated as a dynamic process that evolves over time, rather than a static condition (Figure 2). To strengthen the critical synthesis, we distinguish three levels of support: well-supported (patient education, early troubleshooting, structured follow-up), promising (CBT-I/COMISA, targeted psychosocial interventions), and exploratory (phenotype/endotype-guided titration, advanced adaptive algorithms). Based on consistent RCTs and real-world cohorts, patient education, early troubleshooting, and structured follow-up show the strongest support for improving adherence. Making patients aware of their symptoms, the condition and its consequences is the first element to be considered during patient management. In this phase, reviewing respiratory polygraphy or polysomnographic data with the patient may significantly enhance understanding and engagement. Effective communication and active patient involvement can improve adherence to CPAP. In this review, we have also highlighted how psychosocial factors, including relationship dynamics, mental health disorders and socioeconomic status, can influence treatment acceptance and, consequently, adherence. Insomnia, depression and anxiety can pose significant barriers to treatment adherence if not properly recognized and managed. In this trajectory, cognitive-behavioral interventions in patients with COMISA have demonstrated a significant increase in CPAP adherence [104,105]. In conclusion interventions such as CBT-I in patients with COMISA and programs with psychosocial components appear promising, but heterogeneity of protocols and samples and potential selection bias warrant caution before broad implementation. In the second phase, it is essential to properly introduce CPAP therapy to the patient. This involves not only explaining how it works but also highlighting the potential clinical benefits that can be achieved through CPAP. CPAP titration is a crucial step in determining future adherence to therapy, as it involves the selection of the PAP level and interface. Personalizing the mask choice, optimizing pressure settings and managing initial side effects are essential to ensure comfort and reduce the risk of early discontinuation of therapy. It is important to emphasize that this phase should not be viewed as purely technical. Approaches such as phenotype/endotype-guided titration and advanced adaptive algorithms remain exploratory; multicenter prospective validation and shared operational definitions are needed. In clinical practice, an initial bundle of structured education, a troubleshooting contact within 7–14 days, and planned follow-up offers the best evidence-to-implement ability balance. It is also essential to verify that all residual respiratory events are properly resolved during therapy. The final phase is follow-up. Early follow-up, particularly during the first few weeks of treatment, helps identify patients at higher risk of treatment failure, perhaps even through the use of telemonitoring, which is gaining increasingly strong scientific support. It is important to emphasize that these phases constitute interconnected elements of a multidimensional pathway. The concept highlighted by our review is that adherence to CPAP needs a patient-centered clinical approach (Figure 3).

**Scheme 2:**
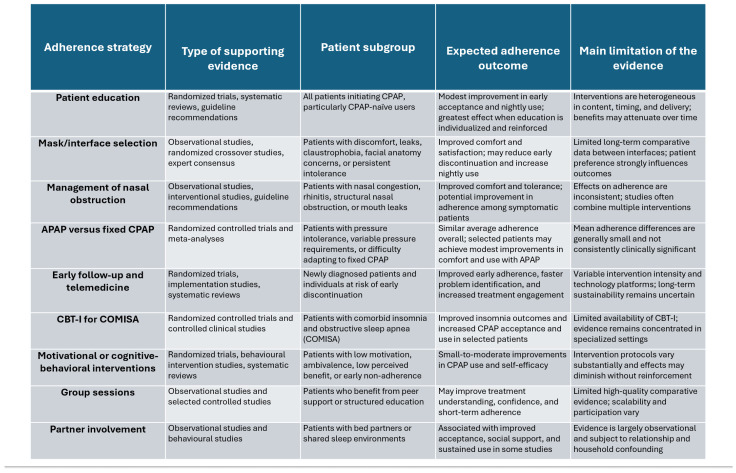
Type of supporting evidence.

This review summarizes many current practical strategies to improve CPAP adherence, and we explicitly classify them to aid interpretation: separating evidence-based recommendations from expert opinion, plausible mechanistic reasoning, and areas where evidence remains preliminary, so readers can gauge the scientific validity of what is reported and see what still requires confirmation (Table 2 and Scheme 2).

## 5. Beyond the Static Model: Dynamics in CPAP Adherence

Although the recent literature emphasizes that adherence is especially variable during the first month—suggesting that, rather than a binary “adherent/non-adherent” distinction, early trajectories or archetypes of adherence can be observed—an underappreciated point remains variability itself [25,106]. Some patients may simply need more time to adapt to CPAP because of physio pathological factors (e.g., high loop gain) or psychological factors (e.g., anxiety) [10,99]. We are adapting CPAP to patients whose sleep states vary across the night and can change once therapy begins (e.g., REM rebound and altered NREM/REM proportions) [107]. A patient with very severe OSA may resume supine sleep they previously avoided; during REM, they may open the mouth; over the night, they may develop an arrhythmia that triggers central events and intolerance. Functional determinants also fluctuate nocturnally: loop gain physiologically tends to rise toward the end of the night, may decrease once CPAP is initiated, and can increase again with intercurrent conditions such as heart failure [50,108]. Likewise, nocturnal fluid redistribution alters upper-airway collapsibility [109]. OSA severity varies not only within a night but also night-to-night. Nevertheless, a person’s underlying endotypic trait remains relatively stable across nights, as suggested by studies on inter-night endotype variability; thus, dynamic fluctuations unfold upon a comparatively consistent endotypic substrate [110]. It is therefore unsurprising that, during the period of adaptation to CPAP and a new breathing condition, these elements can dynamically modify both efficacy and tolerance, underscoring the need for solutions that perform across all nights and throughout each night, and that must be reassessed over time.

Early titration should integrate real-time waveform geometry—flattening, curvature, duty-cycle, breath-level resistance—into a feature-augmented controller that is state-/position-aware, uses shorter response horizons during REM transitions, targets volatile phenotypes (REM-predominant, positional, high loop-gain, low arousal threshold), and runs a week-1/2 feedback review to refine settings before tapering adaptivity [111,112]. The only way to overcome the complexity of each individual’s breathing during sleep and their response to positive airway pressure therapy is the awaited analysis of waveforms and downloaded data [75].

Summary data can give an idea of what is happening, but statistics do not capture human variability. For this reason, during titration, it appears necessary to analyze an adequate number of nights to increase the reproducibility of different conditions (body position or sleep stage) and to perform a manual, critical review of the data. The same applies to follow-up visits, where the ability to see trends over time in compliance, the criticality of certain days compared to others, or the early detection of a factor in non-adherence—such as the reappearance of snoring or flow limitation, or leaks from the mask—represent key aspects of good everyday clinical practice.

## 6. Limitations

This review has some limitations that should be acknowledged. As a narrative review, it does not follow the methodological framework of a systematic review, and the selection of the included studies may therefore have been influenced by the author’s aim to answer necessity in clinical practice. In addition, no formal quality assessment of the included studies was carried out. Consequently, the evidence discussed should be interpreted with caution, particularly in view of the differences in study designs, methodologies, and overall quality. Nevertheless, this review was conceived with a practical clinical focus and was informed, where relevant, by current recommendations from the American Academy of Sleep Medicine. Its added value lies in providing a pragmatic, scientifically grounded overview of the main factors currently limiting CPAP adherence, while also offering practical guidance to support clinicians in addressing this challenging issue.

## 7. Conclusions

This review highlights that CPAP prescription should follow a multidimensional, patient-centered approach that integrates clinical, physiological and educational aspects. Early and personalized patient involvement, personalized education, optimization of therapeutic PAP, mask selection and early management of any initial adverse effects are crucial for short- and long-term adherence. Although technological advances such as new CPAP devices and new interfaces (nasal prongs or under-the-nose masks) have improved patient comfort, these alone are not sufficient to ensure adherence. Instead, a multidimensional approach is required. Patient education regarding the sleep respiratory disorder and CPAP, a tightly scheduled follow-up with prompt troubleshooting, and sustained motivational support constitute the key elements for achieving adherence. Each component of this pathway should be individualized to the patient and calibrated to the operational resources of the sleep center. The role of telemedicine in this regard could be useful to ensuring adherence on a large scale. Our findings do not support telemedicine as a universal solution to ensure adherence, but rather as one of several potential components within structured follow-up pathways. Telemonitoring initiatives are heterogeneous in design and intensity, and their effects may wane over the longer term. Effective implementation depends on adequate infrastructure, integration into clinical workflows, and sound data governance, which can limit scalability in some settings. Suitability also varies across patient groups due to differences in digital access, clinical complexity, and preferences. Future research should clarify which intervention features, delivery models, and patient profiles yield the most sustained benefits, and under what resource conditions. The patient’s psychological profile and social environment have been recognized, in addition to the more extensively researched patient’s treatment and physiological profile. Future research should focus on refining personalized treatment strategies, including the potential role of phenotypic and endotypic characterization, as well as on developing targeted interventions to improve adherence in high-risk populations.

## Figures and Tables

**Table 1:** (**A**). Common barriers to CPAP adherence and prevention strategies. FEMEA approach. (**B**). Common barriers to CPAP adherence and practical management strategies.

(A)						
Failure Mode (Barrier)	Potential Effect	S (1–10)	O (1–10)	D (1–10)	RPN	Proposed Pre-CPAP Mitigation
Low perceived benefit	Loss of motivation → silent discontinuation without prior technical complaint	7	6	7	294	At baseline visit, anchor expectations to the patient’s most bothersome symptom. Some symptoms, like daytime sleepiness, improve gradually with regular CPAP use
Anxiety/depression/claustrophobia	Early interface rejection, discontinuation within the first week	8	7	5	280	Baseline psychological screening; progressive mask desensitization in clinic before the first night at home. Practice with the mask when awake (e.g., afternoon sessions). Add a ramp for easier fall-asleep. Use calming audio: music, podcast, or a brief relaxation tutorial. Start with short trials, then extend to full nights. Simply explain that nasal receptors simply need a normal adjustment period.
Mask leaks	Suboptimal therapeutic pressure, bed-partner disruption, frustration	6	8	4	192	Baseline facial/anatomical assessment (facial hair, absence of teeth, retrognathia) for pre-selection of interface
Pressure intolerance	Mask removal overnight, poor initial adaptation	6	6	5	180	Explicit ramp/EPR setup from night 1, not introduced only as rescue later
Bed-partner conflict	Negative relationship-adherence cycle, device withdrawal under social pressure	5	5	6	150	Include the bed partner in initial education, not only the patient
Dry mouth	Nighttime discomfort, often misattributed to “CPAP intolerance”	4	7	5	140	Phenotype the oral breather at baseline → heated humidification from initiation, not after the complaint
Comorbid insomnia (COMISA)	Prolongs sleep-onset latency with mask on, reinforces rejection	8	6	2	96	CBT-I prior to or concurrent with CPAP initiation. Try and build confidence with the device during the afternoon
Residual events	Incomplete clinical benefit, perceived treatment failure	7	5	2	70	Already readily detectable via telemonitoring—lower priority for preventive action. Sometimes initial ineffectiveness reflects fixable factors (suboptimal pressure, leaks, or interface). Once corrected, efficacy typically follows
Nasal obstruction	Secondary oral breathing, increased resistance, higher pressure requirement	5	4	2	40	ENT history at baseline (rhinitis, septal deviation) already easily screened
**(B)**						
**Barrier to** **CPAP Adherence**	**Practical Management in Routine Care**					**References**
Mask leaks	Reassess mask fit, size, and interface type; avoid over-tightening; replace worn cushions or headgear; consider mask liners or switching to a different interface if leaks persist.					[91]
Nasal obstruction	Evaluate and treat reversible nasal conditions; use saline irrigation, intranasal therapies when indicated, and heated humidification; reserve oronasal masks for selected patients with persistent nasal obstruction.					[100]
Dry mouth	Assess for mouth leaks and ensure appropriate pressure settings, optimize heated humidification; reconsider mask type; consider a chin strap in selected patients; review contributing medications and hydration status.					[91]
Insomnia	Screen for comorbid insomnia (ISI Insomnia Severity Index questionnaire); introduce gradual CPAP acclimatization; use behavioral strategies and CBT-I when available; avoid forcing prolonged use too early if this increases frustration or delays sleep onset.					[101]
Anxiety/depression	Identify claustrophobia or affective symptoms early; use stepwise desensitization; provide reassurance and close early follow-up; refer for psychological support when indicated.					[79]
Low perceived benefit	Reframe expectations regarding the timing and nature of treatment benefits; review adherence and efficacy data with the patient; address side effects; align treatment goals with patient-prioritized outcomes.					[102]
**Bed-partner issues**	Involve the bed partner in patient education; address noise and leak issues; optimize interface comfort; discuss shared benefits such as reduced snoring and improved sleep quality.					[31]
**Residual events**	Review device data for residual AHI, mask leaks, and effective nightly use; reassess pressure settings; consider repeat titration or further evaluation for central events, positional OSA, or other sleep disorders if symptoms persist.					[83]
**Pressure intolerance**	Use ramp and expiratory pressure relief features; adjust pressure settings when poorly tolerated; encourage gradual acclimatization; consider APAP, bilevel or auto bilevel therapy in selected patients.					[103]

**Table 2:** From Evidence-Based to Preliminary: Categorization of Adherence Interventions.

Evidence-Based Recommendations	Expert Opinion		Plausible Mechanistic Reasoning	Preliminary Evidence
Strategy	Evidence Category	Target Subgroup	Expected Outcome	Limitations
Patient education	Evidence-based	Newly diagnosed OSA; all patients	Improved understanding/acceptance; modest increase in nightly use	Heterogeneous content and delivery
Early, structured follow-up and troubleshooting	Evidence-based	First 1–4 weeks; early problems	Reduced dropouts; stabilization of use over time	Resource-intensive; protocol variability
Mask/interface optimization (conditional nasal-first; switch if issues persist)	Evidence-based	No persistent relevant nasal obstruction	Lower leaks; better comfort; signal of improved adherence vs. oronasal	Advantage not universal; mouth breathing may negate benefit
Treat nasal obstruction; optimize humidification	Evidence-based	Rhinitis/congestion/dry mouth	Comfort gains → increased use	Effect depends on underlying nasal disease
Telemedicine/remote monitoring (digital health): routine data review + feedback	Evidence-based	Standard care; early maintenance	Small–moderate adherence increase; earlier issue detection	Modest effect sizes; implementation variability
CBT-I for COMISA (protocolized)	Evidence-based	OSA with comorbid insomnia	Better sleep initiation/maintenance; signal of increased CPAP use	Requires trained staff; not for all phenotypes
Shared decision-making for interface selection	expert opinion	All patients; preference-sensitive	Higher acceptance; fewer early dropouts	Limited RCTs isolating the effect
General psychological support programs (non-protocolized)	Expert opinion	Low motivation/anxiety/claustrophobia	Engagement support; possible increase in persistence	Heterogeneous content; limited controlled data
Chin strap for persistent mouth leaks (selected cases)	Expert opinion	Nasal interface + mouth leaks despite optimization	Fewer leaks/dry mouth symptoms; potential comfort gains	Not universally tolerated; variable benefit
Advanced digital health tools (apps, AI-assisted workflows)	Preliminary	Tech-engaged programs/patients	Early engagement signals; uncertain impact on hours/night	Heterogeneous designs; limited prospective outcomes
Phenotype-/endotype-guided PAP algorithms	Preliminary	Specialized centers; research protocols	Hypothesis-driven personalization; unknown adherence effect	No standardized tools: prospective validation needed
Precision/personalized behavioral interventions beyond CBT-I	Preliminary	Selected behavioral phenotypes	Possible adherence gains in subgroups	Mostly pilot/feasibility data

## Data Availability

No new data were created for this review.

## Supplementary Materials

The following supporting information can be downloaded at: https://www.mdpi.com/article/10.3390/healthcare14152328/s1, Table S1: Full database queries and search details; Table S2: Deduplicated records and screening decisions.
