# Supplementary material for: Optimizing CPAP Adherence in Obstructive Sleep Apnea: A Narrative Review of Practical Strategies for Everyday Clinical Care
Source: Healthcare (Basel). 2026 Aug 1;14(15):2328. doi: 10.3390/healthcare14152328 (PMC13465342; doi:10.3390/healthcare14152328)
Supplement: Supplementary file 1 [file healthcare-14-02328-s001.zip › healthcare-4317926-supplementary.pdf]

Supplementary Table S1 — Full database queries and search details

Source: PubMed/MEDLINE (via PubMed)

Final search date: 2026-06-20

Coverage window: 1995-01-01–2026-06-20

Filters: Humans; Adults 19+ years; English

Exact Boolean queries (as executed)

1) Core adherence to CPAP in OSA

(("Obstructive Sleep Apnea Syndromes"[Mesh] OR "obstructive sleep apnea"[tiab] OR OSA[tiab]) AND ("Continuous Positive Airway Pressure"[Mesh] OR "continuous positive airway pressure"[tiab] OR CPAP[tiab] OR "auto-CPAP"[tiab] OR APAP[tiab]) AND (adherence[tiab] OR compliance[tiab] OR "treatment adherence and compliance"[Mesh])) AND (("1995/01/01"[Date - Publication] : "2026/06/20"[Date - Publication])) AND (humans[mh]) AND (english[lang]) AND (adult[mh])

2) Mask/interface and technical refinements

(("Obstructive Sleep Apnea Syndromes"[Mesh] OR "obstructive sleep apnea"[tiab] OR OSA[tiab]) AND ("Continuous Positive Airway Pressure"[Mesh] OR CPAP[tiab] OR APAP[tiab]) AND ("Masks"[Mesh] OR mask[tiab] OR "mask interface"[tiab] OR "nasal mask"[tiab] OR "oronasal mask"[tiab] OR "full-face mask"[tiab] OR "nasal pillows"[tiab] OR leak[tiab] OR humidification[tiab] OR telemonitor*[tiab] OR telemedicine[tiab])) AND (adherence[tiab] OR compliance[tiab]) AND (("1995/01/01"[Date - Publication] : "2026/06/20"[Date - Publication])) AND (humans[mh]) AND (english[lang]) AND (adult[mh])

3) Phenotype/endotype and adherence

(("Obstructive Sleep Apnea Syndromes"[Mesh] OR "obstructive sleep apnea"[tiab] OR OSA[tiab]) AND (phenotype[tiab] OR "Disease Susceptibility"[Mesh] OR endotype[tiab] OR pathophysiology[tiab] OR "Physiopathology"[Subheading]) AND ("Continuous Positive Airway Pressure"[Mesh] OR CPAP[tiab] OR APAP[tiab]) AND (adherence[tiab] OR compliance[tiab])) AND (("1995/01/01"[Date - Publication] : "2026/06/20"[Date - Publication])) AND (humans[mh]) AND (english[lang]) AND (adult[mh])

4) Female sex/women and adherence

(("Obstructive Sleep Apnea Syndromes"[Mesh] OR "obstructive sleep apnea"[tiab] OR OSA[tiab]) AND (female[tiab] OR women[tiab] OR sex[tiab] OR gender[tiab] OR "Sex Factors"[Mesh]) AND ("Continuous Positive Airway Pressure"[Mesh] OR CPAP[tiab] OR APAP[tiab]) AND (adherence[tiab] OR compliance[tiab])) AND (("1995/01/01"[Date - Publication] : "2026/06/20"[Date - Publication])) AND (humans[mh]) AND (english[lang]) AND (adult[mh])

5) Behavioral interventions and support

(("Obstructive Sleep Apnea Syndromes"[Mesh] OR "obstructive sleep apnea"[tiab] OR OSA[tiab]) AND ("Continuous Positive Airway Pressure"[Mesh] OR CPAP[tiab] OR APAP[tiab]) AND ("Cognitive Behavioral Therapy"[Mesh] OR "cognitive behavioral therapy"[tiab] OR coaching[tiab] OR education[tiab] OR "motivational interview*"[tiab] OR "adherence program*"[tiab] OR support[tiab] OR telemedicine[tiab] OR telemonitor*[tiab])) AND (adherence[tiab] OR compliance[tiab]) AND (("1995/01/01"[Date - Publication] : "2026/06/20"[Date - Publication])) AND (humans[mh]) AND (english[lang]) AND (adult[mh])

6) CPAP vs auto-CPAP and adherence/effectiveness

(("Obstructive Sleep Apnea Syndromes"[Mesh] OR "obstructive sleep apnea"[tiab] OR OSA[tiab]) AND (APAP[tiab] OR "auto-CPAP"[tiab] OR "Automatic Positive Airway Pressure"[Mesh] OR CPAP[tiab]) AND (adherence[tiab] OR compliance[tiab] OR effectiveness[tiab] OR outcomes[tiab])) AND (("1995/01/01"[Date - Publication] : "2026/06/20"[Date - Publication])) AND (humans[mh]) AND (english[lang]) AND (adult[mh])

Supplementary Table S2 — Deduplicated records and screening decisions

Source	Final search date	Title	Authors	Year	Journal	DOI/PMID	Screened title/abstract (Y/N)	Full text assessed (Y/N)	Included (Y/N)	Exclusion reason	Notes
Web	2026-06-20	Adherence to CPAP Treatment and the Risk of Recurrent Cardiovascular Events,	M. Sánchez-de-la-Torre et al.,	2023		10	Y				
Web	2026-06-20	Impact of an early follow-up visit on adherence and adherence trajectories	J. Duval, J. Texereau, S. Bailly, and J. L. Pépin,	2026		10	Y				

Source	Final search date	Title	Authors	Year	Journal	DOI/PMID	Screened title/abstract (Y/N)	Full text assessed (Y/N)	Included (Y/N)	Exclusion reason	Notes
		after initiation of continuous positive airway pressure therapy for obstructive sleep apnea,									
Web	2026-06-20	Adherence to CPAP: What Should We Be Aiming For, and How Can We Get There?,	J. P. Bakker, T. E. Weaver, S. Parthasarathy, and M. S. Aloia,	2019		10	Y				
Web	2026-06-20	Adherence to positive airway pressure therapy and healthcare resource utilization and costs among patients with	F. H. Sert Kuniyoshi et al.,	2026		10	Y				

Source	Final search date	Title	Authors	Year	Journal	DOI/PMID	Screened title/abstract (Y/N)	Full text assessed (Y/N)	Included (Y/N)	Exclusion reason	Notes
		obstructive sleep apnea and obesity,									
Web	2026-06-20	Maximizing positive airway pressure adherence in adults: a common-sense approach,	E. M. Wickwire, C. J. Lettieri, A. A. Cairns, and N. A. Collop,	2013		10	Y				
Web	2026-06-20	Treatment of Adult Obstructive Sleep Apnea with Positive Airway Pressure: An American Academy of Sleep Medicine Clinical Practice Guide-line,	S. P. Patil, I. A. Ayappa, S. M. Caples, R. J. Kimoff, S. R. Patel, and C. G. Harrod,	2019		10	Y				

Source	Final search date	Title	Authors	Year	Journal	DOI/PMID	Screened title/abstract (Y/N)	Full text assessed (Y/N)	Included (Y/N)	Exclusion reason	Notes
Web	2026-06-20	Centers for Medicare and Medicaid Services Positive Airway Pressure Adherence Criteria May Limit Treatment to Many Medicare Beneficiaries,	S. Naik, M. Al-Halawani, I. Kreinin, and M. Kryger,	2019		10	Y				
Web	2026-06-20	The Effect of CPAP in Normalizing Daytime Sleepiness, Quality of Life, and Neurocognitive Function in Patients with Moderate to Severe OSA,	N. A. Antic et al.,	2011		10	Y				

Source	Final search date	Title	Authors	Year	Journal	DOI/PMID	Screened title/abstract (Y/N)	Full text assessed (Y/N)	Included (Y/N)	Exclusion reason	Notes
Web	2026-06-20	REM-OSA as a Tool to Understand Both the Architecture of Sleep and Pathogenesis of Sleep Apnea-Literature Review,	F. F. Karuga et al.,	2023		10	Y				
Web	2026-06-20	REM-associated sleep apnoea: prevalence and clinical significance in the HypnoLaus cohort,	P. Acosta-Castro et al.,	2017		10	Y				
Web	2026-06-20	Effect of rapid eye movement-related obstructive sleep apnea on adherence to	T. Hoshino et al.,	2018		10	Y				

Source	Final search date	Title	Authors	Year	Journal	DOI/PMID	Screened title/abstract (Y/N)	Full text assessed (Y/N)	Included (Y/N)	Exclusion reason	Notes
		continuous positive airway pressure,									
Web	2026-06-20	Incident Type 2 Diabetes in OSA and Effect of CPAP Treatment: A Retrospective Clinic Cohort Study,	P. H. Xu, C. K. M. Hui, M. M. S. Lui, D. C. L. Lam, D. Y. T. Fong, and M. S. M. Ip,	2019		10	Y				
Web	2026-06-20	Acceptance and Compliance of Continuous Positive Airway Pressure (CPAP) Therapy in Patients with Obstructive	S. Subramaniam and A. Raj,	2025		10	Y				

Source	Final search date	Title	Authors	Year	Journal	DOI/PMID	Screened title/abstract (Y/N)	Full text assessed (Y/N)	Included (Y/N)	Exclusion reason	Notes
		Sleep Apnea,									
Web	2026-06-20	Gender differences in obstructive sleep apnea syndrome: a pilot study,	A. Fabozzi et al.,	2024		10	Y				
Web	2026-06-20	Gender differences in obesity hypoventilation syndrome,	E. Barbagelata et al.,	2023		10	Y				
Web	2026-06-20	Age and sex differences in longitudinal CPAP adherence patterns,	M. Vodapally, A. M. Chan-Golston, J. Sandhu, and L. M. DelRosso,	2026		10	Y				
Web	2026-06-20	Sleep apnoea phenotypes in women: A cluster analysis	A. Pataka et al.,	2024		10	Y				

Source	Final search date	Title	Authors	Year	Journal	DOI/PMID	Screened title/abstract (Y/N)	Full text assessed (Y/N)	Included (Y/N)	Exclusion reason	Notes
		from the ESADA cohort,									
Web	2026-06-20	Assessing the needs and perspectives of patients with obesity and obstructive sleep apnea syndrome following continuous positive airway pressure therapy to inform health care practice: A focus group study,	G. Rapelli et al.,	2022		10	Y				
Web	2026-06-20	Three in a Bed: Can Partner Support Improve CPAP	G. Rapelli et al.,	2025		10	Y				

Source	Final search date	Title	Authors	Year	Journal	DOI/PMID	Screened title/abstract (Y/N)	Full text assessed (Y/N)	Included (Y/N)	Exclusion reason	Notes
		Adherence? A Systematic Review and Intervention Recommendations,									
Web	2026-06-20	Factors affecting self-reported sexuality in men with obstructive sleep apnea syndrome,	T. Stannek, C. Hürny, O. D. Schoch, T. Bucher, and T. Münzer,	2009		10	Y				
Web	2026-06-20	Walking the fine line between OSA and aging,	C. Antonaglia, A. Fabozzi, A. Steffanina, R. Ture, P. Palange, and M. Confalonieri,	2025		10	Y				
Web	2026-06-20	Adherence to Positive Airway Pressure Therapy and Healthcare Resource Utilization		2025			Y				

Source	Final search date	Title	Authors	Year	Journal	DOI/PMID	Screened title/abstract (Y/N)	Full text assessed (Y/N)	Included (Y/N)	Exclusion reason	Notes
		and Costs Among Patients with Obstructive Sleep Apnea and Obesity | Research Square.									
Web	2026-06-20	Comorbid Insomnia and Obstructive Sleep Apnea (COMISA): Current Concepts of Patient Management,	B. Ragnoli, P. Pochetti, A. Raie, and M. Malerba,	2021		10	Y				
Web	2026-06-20	Effects of combined morbid insomnia and sleep apnea on long-term cardiovascular risk and all-cause mortality in	F. Fang et al.,	2024		10	Y				

Source	Final search date	Title	Authors	Year	Journal	DOI/PMID	Screened title/abstract (Y/N)	Full text assessed (Y/N)	Included (Y/N)	Exclusion reason	Notes
		elderly patients: a prospective cohort study,									
Web	2026-06-20	Association of Anxiety and Depression in Obstructive Sleep Apnea Patients: A Systematic Review and Meta-Analysis,	S. Garbarino, W. A. Bardwell, O. Guglielmi, C. Chiorri, E. Bonanni, and N. Magnavita,	2018		10	Y				
Web	2026-06-20	Impact of Long-Term CPAP Adherence on Recurrence After Atrial Fibrillation Ablation in Patients With Severe	N. Tanaka et al.,	2025		10	Y				

Source	Final search date	Title	Authors	Year	Journal	DOI/PMID	Screened title/abstract (Y/N)	Full text assessed (Y/N)	Included (Y/N)	Exclusion reason	Notes
		Sleep Apnea,									
Web	2026-06-20	Depression May Reduce Adherence during CPAP Titration Trial,	M. Law, M. Naughton, S. Ho, T. Roebuck, and E. Dabscheck,	2014		10	Y				
Web	2026-06-20	Periodic limb movements during sleep are associated with poor health-related quality of life in patients with obstructive sleep apnea | Sleep and Breathing | Springer Nature Link.		2026		10.1007/s11325-021-02469-y	Y				

Source	Final search date	Title	Authors	Year	Journal	DOI/PMID	Screened title/abstract (Y/N)	Full text assessed (Y/N)	Included (Y/N)	Exclusion reason	Notes
Web	2026-06-20	Sleep microstructure and clinical characteristics of patients with restless legs syndrome,	H. Amirifard et al.,	2022		10	Y				
Web	2026-06-20	Phenotypic Subtypes of OSA: A Challenge and Opportunity for Precision Medi-cine,	A. Zinchuk and H. K. Yaggi,	2019		10	Y				
Web	2026-06-20	Positive Nasal Airway Pressure Eliminates Snoring As Well As Obstructive Sleep Apnea,	R. B. Berry and A. J. Block,	1984		10	Y				

Source	Final search date	Title	Authors	Year	Journal	DOI/PMID	Screened title/abstract (Y/N)	Full text assessed (Y/N)	Included (Y/N)	Exclusion reason	Notes
Web	2026-06-20	OSA and CPAP therapy: effect of gender, somnolence, and treatment adherence on health-related quality of life,	A. Lo Bue, A. Salvaggio, S. Iacono Isidoro, S. Romano, and G. Insalaco,	2020		10	Y				
Web	2026-06-20	Sex differences in low arousal threshold in obstructive sleep apnea,	G. M. Citton et al.,	2025		10	Y				
Web	2026-06-20	Phenotypic approaches to obstructive sleep apnoea – New pathways	D. J. Eckert,	2016		10	Y				

Source	Final search date	Title	Authors	Year	Journal	DOI/PMID	Screened title/abstract (Y/N)	Full text assessed (Y/N)	Included (Y/N)	Exclusion reason	Notes
		for targeted therapy,									
Web	2026-06-20	Defining phenotypic causes of obstructive sleep apnea. Identification of novel therapeutic targets,	D. J. Eckert, D. P. White, A. S. Jordan, A. Malhotra, and A. Wellman,	2013		10	Y				
Web	2026-06-20	A simplified method for determining phenotypic traits in patients with obstructive sleep apnea,	A. Wellman et al.,	2012		10	Y				
Web	2026-06-20	Clinical characteristics of Asian patients with sleep apnea with low arousal threshold and sleep	L.-W. Hang, C.-S. Huang, and W.-J. Cheng,	2021		10	Y				

Source	Final search date	Title	Authors	Year	Journal	DOI/PMID	Screened title/abstract (Y/N)	Full text assessed (Y/N)	Included (Y/N)	Exclusion reason	Notes
		structure change with continuous positive airway pressure,									
Web	2026-06-20	Clinical predictors of the respiratory arousal threshold in patients with obstructive sleep apnea,	B. A. Edwards et al.,	2014		10	Y				
Web	2026-06-20	Deciphering loop gain complexity: a primer for understanding a pathophysiological trait of obstructive sleep apnea patients,	C. Antonaglia et al.,	2024		10	Y				

Source	Final search date	Title	Authors	Year	Journal	DOI/PMID	Screened title/abstract (Y/N)	Full text assessed (Y/N)	Included (Y/N)	Exclusion reason	Notes
Web	2026-06-20	Auto-adjusting positive airway pressure: the fine line between engineering and medicine | Sleep and Breathing | Springer Nature Link.		2026		10.1007/s11325-025-03409-w	Y				
Web	2026-06-20	Research Priorities for Translating Endophenotyping of Adult Obstructive Sleep Apnea to the Clinic: An Offi-cial American Thoracic Society Research Statement | American		2026			Y				

Source	Final search date	Title	Authors	Year	Journal	DOI/PMID	Screened title/abstract (Y/N)	Full text assessed (Y/N)	Included (Y/N)	Exclusion reason	Notes
		Journal of Respiratory and Critical Care Medicine ǀ Oxford Academic.									
Web	2026-06-20	Equivalence of Nasal and Oronasal Masks during Initial CPAP Titration for Obstructive Sleep Apnea Syndrome,	M. Teo, T. Amis, S. Lee, K. Falland, S. Lambert, and J. Wheatley,	2011		10	Y				
Web	2026-06-20	In search of a better CPAP interface: A network meta-analysis comparing nasal masks, nasal pillows and	L.-Y. Chen et al.,	2022		10	Y				

Source	Final search date	Title	Authors	Year	Journal	DOI/PMID	Screened title/abstract (Y/N)	Full text assessed (Y/N)	Included (Y/N)	Exclusion reason	Notes
		oronasal masks,									
Web	2026-06-20	Partitioning of inhaled ventilation between the nasal and oral routes during sleep in normal subjects | Journal of Applied Physiology | American Physiological Society.		2002		10.1152/japplphysiol.00658.2002	Y				
Web	2026-06-20	Effect of nasal or oral breathing route on upper airway resistance during sleep,	M. F. Fitzpatrick, H. McLean, A. M. Urton, A. Tan, D. O'Donnell, and H. S. Driver,	2003		10	Y				
Web	2026-	Impact of the type of mask on the	R. G. S. de Andrade, V. S. Piccin, J. A. Nascimento, F. M. L.	20		10	Y				

Source	Final search date	Title	Authors	Year	Journal	DOI/PMID	Screened title/abstract (Y/N)	Full text assessed (Y/N)	Included (Y/N)	Exclusion reason	Notes
	06-20	effectiveness of and adherence to continuous positive airway pressure treatment for obstructive sleep apnea,	Viana, P. R. Genta, and G. Lorenzi-Filho,	14							
Web	2026-06-20	Young, J., Finn, L. and Kim, H. (1997) Nasal Obstruction as a Risk Factor for Sleep-Disordered Breathing. The Journal of Allergy and Clinical Immunology, 99, S757-S762. - References - Scientific		2025			Y				

Source	Final search date	Title	Authors	Year	Journal	DOI/PMID	Screened title/abstract (Y/N)	Full text assessed (Y/N)	Included (Y/N)	Exclusion reason	Notes
		Research Publishing.									
Web	2026-06-20	Nasal pillow vs. standard nasal mask for treatment of OSA: a systematic review and me-ta-analysis,	B. Deng et al.,	2023		10	Y				
Web	2026-06-20	Breathing route influences upper airway muscle activity in awake normal adults,	R. C. Basner, P. M. Simon, R. M. Schwartzstein, S. E. Weinberger, and J. W. Weiss,	1989		10	Y				
Web	2026-06-20	Influence of Nasal Resistance on Initial Acceptance of Continuous	T. Sugiura et al.,	2005		10	Y				

Source	Final search date	Title	Authors	Year	Journal	DOI/PMID	Screened title/abstract (Y/N)	Full text assessed (Y/N)	Included (Y/N)	Exclusion reason	Notes
		Positive Airway Pressure in Treatment for Obstructive Sleep Apnea Syndrome,									
Web	2026-06-20	The effects of topical nasal steroids on continuous positive airway pressure compliance in patients with obstructive sleep ap-nea: a systematic review and meta-analysis,	N. Charakorn, P. Hirunwiwatkul, N. Chirakalwasan, B. Chaitusaney, and M. Prakassajjatham,	2017		10	Y				
Web	2026-	Auto-adjusting Positive Airway				10	Y				

Source	Final search date	Title	Authors	Year	Journal	DOI/PMID	Screened title/abstract (Y/N)	Full text assessed (Y/N)	Included (Y/N)	Exclusion reason	Notes
	06-20	Pressure Treatment for Sleep Apnea Diagnosed by Home Sleep Testing | Re-quest PDF,									
Web	2026-06-20	Factors associated with treatment outcomes after use of auto-titrating CPAP therapy in adults with obstructive sleep apnea | Sleep and Breathing | Springer Nature Link.		2026		10.1007/s11325-022-02590-6	Y				

Source	Final search date	Title	Authors	Year	Journal	DOI/PMID	Screened title/abstract (Y/N)	Full text assessed (Y/N)	Included (Y/N)	Exclusion reason	Notes
Web	2026-06-20	How Early in Treatment is PAP Adherence Established? Revisiting Night-to-Night Variability: Behavioral Sleep Medicine: Vol 5 , No 3 - Get Access.		2026		10.1080/15402000701264005	Y				
Web	2026-06-20	Trends in CPAP adherence over twenty years of data collection: a flattened curve | Journal of Otolaryngology - Head & Neck Surgery | Springer		2026		10.1186/s40463-016-0156-0	Y				

Source	Final search date	Title	Authors	Year	Journal	DOI/PMID	Screened title/abstract (Y/N)	Full text assessed (Y/N)	Included (Y/N)	Exclusion reason	Notes
		Nature Link.									
Web	2026-06-20	Differences in Perceptions of the Diagnosis and Treatment of Obstructive Sleep Apnea and Continuous Positive Airway Pressure Therapy Among Adherers and Nonadherers - PMC.		2026			Y				
Web	2026-06-20	Polysomnograph chart view by patients: a new educational strategy to	V. A. Falcone, M. F. Damiani, V. N. Quaranta, A. Capozzolo, and O. Resta,	2014		10	Y				

Source	Final search date	Title	Authors	Year	Journal	DOI/PMID	Screened title/abstract (Y/N)	Full text assessed (Y/N)	Included (Y/N)	Exclusion reason	Notes
		improve CPAP adherence in sleep apnea therapy,									
Web	2026-06-20	‘The terrible dryness woke me up, I had some trouble breathing’—Critical situations related to oral health as described by CPAP-treated persons with obstructive sleep apnea - Ahonen - 2022 - Journal of Sleep Research - Wiley		2026		10.1111/jsr.13670	Y				

Source	Final search date	Title	Authors	Year	Journal	DOI/PMID	Screened title/abstract (Y/N)	Full text assessed (Y/N)	Included (Y/N)	Exclusion reason	Notes
		Online Library.									
Web	2026-06-20	Side effects to continuous positive airway pressure treatment for obstructive sleep apnoea: changes over time and association to adherence | Sleep and Breathing | Springer Nature Link.		2026		10.1007/s11325-014-0945-5	Y				
Web	2026-06-20	Practical Aspects of Interface Application in CPAP Treatment -		2019		10.1155/2019/7215258	Y				

Source	Final search date	Title	Authors	Year	Journal	DOI/PMID	Screened title/abstract (Y/N)	Full text assessed (Y/N)	Included (Y/N)	Exclusion reason	Notes
		Bachour - 2019 - Canadian Respiratory Journal - Wiley Online Library.									
Web	2026-06-20	Effects of telemedicine interventions in improving continuous positive airway pressure adherence in patients with obstructive sleep apnoea: a meta-analysis of randomised controlled trials	Sleep and Breathing	Springer		2026		10.1007/s11325-021-02292-5	Y				

Source	Final search date	Title	Authors	Year	Journal	DOI/PMID	Screened title/abstract (Y/N)	Full text assessed (Y/N)	Included (Y/N)	Exclusion reason	Notes
		Nature Link.									
Web	2026-06-20	Increased Adherence to CPAP With a Group Cognitive Behavioral Treatment Intervention: A Randomized Trial | SLEEP | Oxford Academic.		2026			Y				
Web	2026-06-20	The presence of insomnia and depression contributes to the acceptance of an initial treatment trial of contin-uous positive		2026			Y				

Source	Final search date	Title	Authors	Year	Journal	DOI/PMID	Screened title/abstract (Y/N)	Full text assessed (Y/N)	Included (Y/N)	Exclusion reason	Notes
		airway pressure therapy in patients with obstructive sleep apnea - PubMed.									
Web	2026-06-20	Anxiety and depressive symptoms on continuous positive airway pressure: long-term adherence in patients with sleep apnea syndrome - Minerva Medica 2022 December;113(6):967-73.		2026			Y				
Web	2026-	(PDF) Practical Aspects of Interface		20		10	Y				

Source	Final search date	Title	Authors	Year	Journal	DOI/PMID	Screened title/abstract (Y/N)	Full text assessed (Y/N)	Included (Y/N)	Exclusion reason	Notes
	06-20	Application in CPAP Treatment,		19							
Web	2026-06-20	Nasal function and CPAP compliance,	A. Inoue, S. Chiba, K. Matsuura, H. Osafune, R. Capasso, and K. Wada,	2018		10	Y				
Web	2026-06-20	Adherence to CPAP Treatment: Can Mindfulness Play a Role?		2075			Y				
Web	2026-06-20	Effects of Insomnia and Depression on CPAP Adherence in a Military Population - PMC.		2026			Y				
Web	2026-06-20	Psychological predictors of CPAP therapy adherence in	V. Poletti, E. Battaglia, and E. Volpato,	2025		10	Y				

Source	Final search date	Title	Authors	Year	Journal	DOI/PMID	Screened title/abstract (Y/N)	Full text assessed (Y/N)	Included (Y/N)	Exclusion reason	Notes
		obstructive sleep apnea patients: insights from the predisposing, precipitating, and perpetuating factors model,									
Web	2026-06-20	Three in a Bed: Can Partner Support Improve CPAP Adherence? A Systematic Review and Intervention Recommendations,	G. Rapelli et al.,	2025		10	Y				
Web	2026-06-20	Residual Events during Use of CPAP: Prevalence,	J. Reiter, B. Zleik, M. Bazalakova, P. Mehta, and R. J. Thomas,	2016		10	Y				

Source	Final search date	Title	Authors	Year	Journal	DOI/PMID	Screened title/abstract (Y/N)	Full text assessed (Y/N)	Included (Y/N)	Exclusion reason	Notes
		Predictors, and Detection Accuracy,									
Web	2026-06-20	Positive airway pressure adherence and sub-threshold adherence in posttraumatic stress disorder patients with comorbid sleep apnea,	B. J. Krakow, J. J. Obando, V. A. Ulibarri, and N. D. McIver,	2017		10	Y				
Web	2026-06-20	Patterns and stability of long-term adherence in continuous positive airway pressure therapy for obstructive		2026		10.1007/s11325-025-03418-9	Y				

Source	Final search date	Title	Authors	Year	Journal	DOI/PMID	Screened title/abstract (Y/N)	Full text assessed (Y/N)	Included (Y/N)	Exclusion reason	Notes
		sleep apnea: a cohort study	Sleep and Breathing	Springer Nature Link.									
Web	2026-06-20	Increased Adherence to CPAP With a Group Cognitive Behavioral Treatment Intervention: A Randomized Trial	SLEEP	Oxford Academic.		2026			Y				
Web	2026-06-20	Association between age and comorbid insomnia	M. Wu, P. Xue, J. Yan, and C. Benedict,	2024		10	Y				

Source	Final search date	Title	Authors	Year	Journal	DOI/PMID	Screened title/abstract (Y/N)	Full text assessed (Y/N)	Included (Y/N)	Exclusion reason	Notes
		and sleep apnea,									
